# Unraveling the Role of Innate Lymphoid Cells in Acute Myeloid Leukemia

**DOI:** 10.3390/cancers13020320

**Published:** 2021-01-17

**Authors:** Matthew R. Lordo, Steven D. Scoville, Akul Goel, Jianhua Yu, Aharon G. Freud, Michael A. Caligiuri, Bethany L. Mundy-Bosse

**Affiliations:** 1 Biomedical Sciences Graduate Program, Medical Scientist Training Program, Columbus, OH 43210, USA; Matthew.Lordo@osumc.edu; 2 Comprehensive Cancer Center, The Ohio State University, Columbus, OH 43210, USA; Steven.Scoville@osumc.edu (S.D.S.); Aharon.Freud@osumc.edu (A.G.F.); 3 Department of Surgery, The Ohio State University, Columbus, OH 43210, USA; 4 Department of Hematology & Hematopoietic Cell Transplantation, City of Hope National Medical Center, Los Angeles, CA 91010, USA; akulgoel6@gmail.com (A.G.); jiayu@coh.org (J.Y.); 5 Department of Pathology, The Ohio State University Wexner Medical Center, Columbus, OH 43210, USA; 6 Division of Hematology, Department of Internal Medicine, The Ohio State University Wexner Medical Center, Columbus, OH 43210, USA

**Keywords:** AML, ILC, immunotherapy, AHR, NK

## Abstract

**Simple Summary:**

Acute myeloid leukemia (AML) is an aggressive form of cancer found in the blood and bone marrow with poor survival rates. Patients with AML are known to have many defects in their immune system which render immune cells unable to detect and/or kill cancer cells. Natural Killer (NK) cells are innate immune effector cells responsible for surveying the body to eliminate cancer cells as well as alert other immune cells to help clear the cancer cells. NK cells have developmental and functional defects in AML patients. While advances have been made to understand these NK cell defects in the setting of AML, the role of other closely related and recently discovered members of the innate lymphoid cell (ILC) family is much less clear. The ILC family is comprised of NK cells, ILC1s, ILC2s, and ILC3s, and due in part to their recent discovery, non-NK ILCs are just now beginning to be investigated in the setting of AML. By better understanding how AML alters the normal function of these cell types, and how the alteration regulates AML growth, we may be able to target and tailor new forms of therapy for patients.

**Abstract:**

Over the past 50 years, few therapeutic advances have been made in treating acute myeloid leukemia (AML), an aggressive form of blood cancer, despite vast improvements in our ability to classify the disease. Emerging evidence suggests the immune system is important in controlling AML progression and in determining prognosis. Natural killer (NK) cells are important cytotoxic effector cells of the innate lymphoid cell (ILC) family that have been shown to have potent anti-leukemic functions. Recent studies are now revealing impairment or dysregulation of other ILCs in various types of cancers, including AML, which limits the effectiveness of NK cells in controlling cancer progression. NK cell development and function are inhibited in AML patients, which results in worse clinical outcomes; however, the specific roles of other ILC populations in AML are just now beginning to be unraveled. In this review, we summarize what is known about the role of ILC populations in AML.

## 1. Introduction

Acute myeloid leukemia (AML) is a hematologic malignancy marked by unregulated clonal proliferation and infiltration of immature myeloblasts in the bone marrow, blood, and other tissues with overall 5-year survival rates of <28%, which have remained largely unchanged despite marked improvements in our understanding of its pathogenesis [1]. AML is known for considerable cytogenetic and molecular heterogeneity leading to variable disease progression and responses to therapy [2]. The mainstay of treatment has relied on chemotherapy and allogeneic hematopoietic stem cell transplantation (alloHSCT) with marginal improvements over the past several decades [3,4,5]. Immunotherapy beyond alloHSCT for AML has provided some new opportunities beyond traditional forms of therapy [6,7]. NK cell therapies in particular showed promising results following alloHSCT or adoptive NK cell therapy from haploidentical killer immunoglobulin receptor (KIR) mismatched donors [8,9,10,11,12]. Consequently, considerable effort has been invested in trying to understand the cellular and molecular underpinnings that allow AML to thwart these innate effector cells. Research over the last 15 years has revealed that NK cells belong to a larger class of innate lymphoid cells (ILCs) that resemble T cell subsets. In this review, we will summarize the body of recent literature describing how these ILC populations function in the setting of AML and how some of these populations may have therapeutic efficacy in treating AML.

ILCs consist of three groups of cells identified by specific compilations of cell surface markers, transcription factors, and ultimately function [13]. Group 1 ILCs include NK cells as well as type 1 innate lymphoid cells (ILC1s). Both NK cells and ILC1s express the transcription factor *TBX21* (T-BET), which regulates expression of type 1 cytokines including interferon gamma (IFNγ). However, only NK cells co-express the transcription factor *Eomesodermin* (EOMES), which regulates expression of cytolytic perforins and granzymes [14]. Thus, NK cells possess cytolytic effector functions whereas ILC1s are more immunomodulatory and classically lack the ability to directly lyse target cells, although some reports have suggested they may possess weak cytotoxic function [15,16]. Group 2 ILCs consists of ILC2s, which express high levels of the transcription factor GATA3 and secrete type 2 cytokines, such as IL-4, IL-5, and IL-13, as well as molecules such as amphiregulin (AREG). Group 3 ILCs are a heterogeneous subgroup of the ILC family comprised of ILC3s and lymphoid tissue inducer cells (LTIs). All ILC3s express the transcription factor *RORC* (RORγt) and secrete IL-17 and IL-22. A more comprehensive discussion of these ILCs can be found in these review articles [13,17,18]. In this review, we provide updates on recent advances in the field that further our understanding of how AML affects each of the members of the ILC family (summarized in Table 1).

## 2. NK Cells: Key Innate Immune Surveyors

Since their discovery more than 50 years ago, NK cells have been extensively studied for their ability to detect and kill malignant or virally infected cells without prior exposure to specific stimuli. Since then, many groups have described the anti-cancer effects of NK cells in multiple malignancies, including in AML [12,19,36]. The discovery in the early 2000s by Ruggeri et al. that haplo-mismatched donor NK cells can lead to alloreactive responses and improve survival in AML patients treated with alloHSCT launched NK cells towards the forefront as a potential candidate for immunotherapy [8].

More recent studies have continued to define how NK cells develop and function in the setting of AML. NK cells begin their development in the bone marrow and complete their maturation in secondary lymphoid tissues such as the tonsil or in lymph nodes [37,38,39]. The majority of circulating NK cells are mature CD56^dim^ NK cells, and a minority are CD56^bright^ NK cells; however, less mature NK cell precursor populations can also be detected in the circulation at low levels [40]. In contrast to this normal developmental pattern, several groups have demonstrated NK cell maturation defects in patients with AML. We have previously demonstrated a blockade in NK cell development in mouse models of AML marked by failure to progress from stage 2 (CD27+CD11b−) to stage 3 (CD27+CD11b+) NK cells in the spleen [19]. This developmental blockade was also demonstrated using human ILC precursors, which failed to mature into NK cells in the presence of AML cells utilizing ex vivo co-culture models [20]. Importantly, in both of these studies, these observations were reversible, suggesting therapeutic targeting of this pathway is possible to restore mature effector NK cells. It has also been shown that AML patients have significantly lower proportions of circulating NK cells compared to healthy subjects that correlates with a worse prognosis [41]. In addition to reduced frequencies of NK cells, Chretien et al. demonstrated that AML patients often have a hypomature circulating NK cell phenotype, measured by CD57 and KIR expression, and had worse overall survival than patients possessing a more mature NK cell profile in their peripheral blood and tissues [21]. While some AML patients have been found to have a hypomature NK cell profile, others have a hypermature phenotype, defined by expression of CD57, KIR, and dim expression of CD56 [22]. Of note, these latter two studies did not directly assess NK cell function. It is possible that both the hypo- and hypermature phenotypes have similar functional deficiencies as the hypermature cells may represent an exhausted state unable to mount an immune response to cancer targets. Further study is needed to fully characterize these phenotypes and their interactions with AML. 

While not described in the context of NK cell development, studies of NK cells in AML patients have demonstrated impaired NK cell function with an increase in NK cells that express high levels of the inhibitory receptor NKG2A at the time of diagnosis [27]. Similarly, AML patients with reduced expression of NK cell activating receptors such as NKp46 or NKp30 also have worse clinical outcomes [28,29], while patients expressing higher amounts of these receptors have better outcomes [30,31]. Patients with myelodysplastic syndrome (MDS), often a precursor to AML, exhibit NK cell defects as well. Reduced NK cell numbers and decreased functionality resulting from decreased surface expression of activating receptors such as NKp30 or NKG2D have been described in MDS [42,43]. Expectedly, the degree of inhibition of both NK cell frequency and function in MDS appears to be intermediate between healthy individuals and patients with AML. Furthermore, MDS patients with more significant NK dysfunction have higher rates of progression to AML [42].

Additional studies have also described genetic predispositions to developing MDS and AML with concomitant NK cell dysfunction. Genetic loss of the transcription factor GATA2 not only predisposes individuals to MDS/AML [44,45], but also results in profound NK cell defects, namely loss of CD56^bright^ cells with retention of the CD56^dim^ subset [46]. This NK cell defect is present even in patients without MDS or AML who still possess loss of function GATA2 mutations. Interestingly, this observation in nonleukemic individuals phenotypically overlaps with peripheral NK populations in AML patients without GATA2 mutations [19]. This suggests that the GATA2 pathway may play an important role in maintaining NK cell homeostasis in the setting of AML. Studying NK cell and ILC defects in other genetic predisposition syndromes is an area of active investigation.

Although NK cells appear globally inhibited at the time of diagnosis in AML patients, their role in the post-remission setting has revealed they play a critical role in preventing or delaying relapse [6,47,48,49]. Following chemotherapy induction, there is a rapid reconstitution of the NK cell compartment that is generally skewed towards immature CD56^bright^ cells for at least 4 months after, and up to 1 year following, first remission [50]. Patients displaying impaired NK cell function at diagnosis show at least partial restoration of NK cell function at remission, measured by increased activating NKp46 surface expression and increased CD107a expression upon exposure to K562 targets [27]. Studies have also demonstrated therapeutic interventions capable of inducing surface expression of other activating receptors that can further improve the capacity of NK cells to target and kill leukemic targets, including treatment with IL-15 [51]. These studies suggest NK cell functional defects are at least partially reversible, indicating that further research may be able to optimize endogenous NK cell function in AML patients. Overall, NK cell function closely correlates with clinical outcomes in AML patients, but whether this correlation translates to “cause and effect” is still under investigation. 

## 3. NK Cell Therapies in AML

In recent years, adoptive cell therapy utilizing allogeneic NK cells has gained traction as a potential anticancer therapy, especially as a bridge to alloHSCT for AML patients and even as a treatment in patients with relapsed or refractory disease. One such study by Björklund et al., evaluated the effectiveness of IL-2-activated allogeneic NK cells in treating 16 AML patients with refractory disease (median age: 64 years, range: 40–70 years) [11]. The NK cells were derived from human leukocyte antigen (HLA) haploidentical donor peripheral blood mononuclear cells (PBMCs) depleted for T and B cells, and the patients were administered a lymphodepleting regimen consisting of fludarabine/cyclophosphamide conditioning combined with total lymphoid irradiation before treatment. Six of the 16 patients achieved complete remission (CR), marrow CR (mCR), or partial remission (PR). Of these, 5 of the 6 patients proceeded to alloHSCT treatment. Three patients were able to achieve durable clinical responses and survived >3 years after treatment. Notably, 2 additional patients achieved stable disease, and another reached morphological disease-free survival for >3 years after administration of this treatment. These data further support the notion that NK cells may have a direct anti-leukemic effect on AML, even in patients who are otherwise chemotherapy resistant. Dolstra et al. evaluated the effectiveness of an NK cell therapy in 10 older AML patients (median age: 72, range: 68–76 years), all of whom had achieved morphologic CR following induction chemotherapy but had contraindications for alloHSCT [52]. The NK cells were derived and expanded ex vivo from CD34+ hematopoietic stem and progenitor cells (HSPC), which were found in HLA-matched umbilical cord blood (UCB). In this study, 4 of the 10 AML patients were alive at the conclusion of the trial while the other 6 patients relapsed at a mean duration of 364 days after NK cell infusion. Interestingly, the HSPC derived NK cells continued to mature in vivo following transplant, measured by KIR and CD16 acquisition in vivo. Overall, these trials demonstrated the safety profile and potential promise of NK cell-based therapy, and importantly, few side effects >grade 2 were reported. As side effects of adoptive CAR T cell therapies have been a major rate limiting component of treatments [53], these early studies suggest adoptive NK cell therapy is safe in certain subsets of AML patients, with relatively low risk of severe adverse side effects. Future studies will need to determine the clinical efficacy of these adoptive therapies in treating AML patients.

To begin to understand the complex interactions between adoptive NK cells and AML cells, a study by Zhao et al. explored NK cell education following haplo-HSCT transplant in AML and MDS patients [54]. NK cell education and licensing are a process whereby NK cells interact with cognate inhibitory ligands via their corresponding receptors, allowing for variable reactivity to future stimuli and sensitivity to inhibition [55]. These authors discovered that the highest reconstitution of NK cells in patients post-transplant correlated with the lowest relapse rates in patients and occurred when both the donor and host expressed the same KIR ligands and had all the corresponding KIR receptors for those ligands. Expression of inhibitory KIR on donor NK cells and expression of cognate ligands on host cells correlated with better donor NK cell education and therefore reduced frequencies of post-transplant relapse in these patients. This contrasts with the work of Ruggeri et al. [8], where HLA mismatch and/or KIR incompatibly was beneficial in generating alloreactive responses in patients. These seemingly contradictory findings suggest there is a balance between proper NK cell education/licensing through compatible inhibitory KIR interactions and having enough mismatch to generate alloreactive responses important in the graft vs. leukemia effect. In contrast to both of these studies, studies in pediatric populations have found no association between KIR compatibility and clinical outcome [56]. Thus, more work must clearly be done to try and understand the dynamic interactions between donor KIR and host immune cells.

Building upon adoptive cellular therapies, an emerging therapeutic modality in the treatment of malignancies such as AML is chimeric antigen receptor (CAR)-engineered T or NK cells. This form of therapy utilizes genetic engineering to create CAR that possess antigen-specific extracellular domains tethered to intracellular signaling domains that, upon recognition of a tumor associated antigen, can activate downstream signaling pathways to kill tumor cells [57]. Thus far, CAR T cells have proven to be effective in the setting of relapsed/refractory, CD19-expressing B cell malignancies such as acute lymphoblastic leukemia (ALL) and diffuse large B cell lymphoma (DLBCL) [58]. However, the use of CAR T cells has yet to be widely effective against AML, likely due to the heterogeneity of the disease, narrow therapeutic window, side effects of CAR T therapy, and the difficulty in identifying a common surface antigen to target [59]. Clinical trials using CAR NK cells have demonstrated low rates of infusion reactions and therapy-related cytokine release syndrome, side effects commonly encountered in CAR T cell therapy. The first phase 1 study utilizing CD33-CAR NK-92 cells in relapsed/refractory AML patients did not detect dose limiting toxicities at up to 5 billion cells per patient [60]. One limitation of using NK-92 cells, which are a cancer cell line, is that they must be irradiated to limit proliferation in the recipient. This results in a short half-life and thus repeated dosing is likely required to show durable responses [60]. However, the use of these CAR NK-92 cells more readily favors an “off the shelf” CAR therapy for cancer patients that can be scaled into mass production and thus can be delivered at a much lower cost than with current CAR T cell therapies. Rather than irradiation, future work may attempt to engineer genetic kill switches into the NK-92 cells to control their in vivo proliferation. More clinical trials are needed utilizing these CAR NK cells to determine their clinical efficacy in improving disease control following relapse.

Other forms of NK cell therapies currently being tested for their clinical efficacy in AML include deriving NK cells from induced pluripotent stem cells (iPSC) and/or expanding blood NK cells ex vivo utilizing K562 feeder cells. A phase 1 trial utilizing NK cells expanded ex vivo using membrane-bound IL21 (mbIL21) expressing K562 feeder cells found low incidences of infusion-related reactions, although some patients acquired mild GVHD related symptoms [10]. Of the 13 patients included in the study, only one relapsed while the others were in remission at last follow-up (median: 14.7 months). Utilizing this membrane bound cytokine allowed for improved expansion and in vivo persistence over previous methods [61]. The promising results from this study have also led to the formation of a phase 2 trial, which will investigate CSTD002, a product derived from haploidentical donor NK cells expanded ex vivo utilizing PM21 nanoparticles containing mbIL21 and 4-1BBL [62]. Additionally, studies of iPSC-derived, K562-expanded, anti-CD19 CAR-NK cells have shown clinical efficacy in CD19-expressing lymphoid malignancies [63]. As our ability to target AML cells with engineered CAR-NK cells improves, it will be important to improve persistence and expansion likely utilizing membrane bound cytokines and a robust feeder cell platform, respectively. Similarly, studies utilizing iPSC-derived NK cells lacking CISH, a negative regulator of IL-15 signaling, have demonstrated in vivo efficacy in preclinical mouse models of AML that will hopefully lead to phase 1 clinical trials [64].

## 4. Memory-Like NK Cells in AML

Although NK cells are members of the innate immune system, evidence in mice and humans suggests that NK cells can acquire memory-like or adaptive properties following cytomegalovirus (CMV) infection or IL-12/15/18 pre-activation [65]. These stimuli have been shown to induce epigenetic reprogramming of the NK cells resulting in long-term memory-like qualities which are now being studied for potential therapeutic efficacy. In humans, this population is identified by expression of the NKG2C activating receptor and by a response to stimuli with higher levels of IFNγ secretion compared to naïve counterparts. In AML this has been shown to be particularly relevant, as patient’s with CMV reactivation following induction chemotherapy in AML had expansion of this memory-like population which was associated with longer periods of relapse free survival compared to CMV- patients, although overall survival was not significantly altered [23,24,25]. More recently, the anti-leukemia properties of cytokine induced memory-like (CIML) NK cells have been studied as a potential immunotherapy in AML. These CIML NK cells had increased IFNγ and granzyme production upon exposure to AML cells and some patients with relapsed/refractory AML were able to achieve clinical remissions following in fusion with CIML NK cells [66]. Pre-stimulation with IL-12/15/18 has also been shown to upregulate CD25, a component of the heteromeric high affinity IL-2 receptor, which appears to allow CIML NK cells to outcompete inhibitory Treg cells that express this receptor and otherwise act to suppress anti-cancer immune reactions [67,68]. IL-2 is often required for adoptive cellular therapies to increase persistence of the transplanted cells in the host [12]. Recently, a clinical trial utilizing exogenous IL-15 rather than IL-2 with adoptive NK therapy demonstrated some promise in patients with refractory AML and did not stimulate Treg populations [69]. However, some patients experienced cytokine release syndrome symptoms and neurotoxicity, suggesting optimization of dosing route and schedule is required to mitigate these side effects. Newer generation therapies are trying to lessen the need for exogenous cytokine injections by engineering cells to include autocrine signaling mechanisms [70]. This will hopefully reduce the chances for simultaneous stimulation of host Treg populations and specifically bolster the anti-cancer immune response.

## 5. ILC1s: Shedding Light on the Enigma

While NK cells have been well documented as anti-cancer effector cells, a functional role for other ILCs in cancer has yet to be widely established. Initial work has demonstrated the importance of ILC1s in promoting anti-viral immunity in mice [71]. However, their importance in AML remains unclear and is an area of active investigation. Part of the challenge associated with studying ILC1s is a lack of unique positive markers to assist in their identification and thus they are often described as lacking specific NK, ILC2, and ILC3 specific markers [72]. An early report looking at the peripheral blood and marrow of AML patients showed that null definition ILC1s (Lin-CD56-CD127+CRTH2-CD117-) are enriched and hypofunctional compared to healthy donors [32]. A more recent report described an ILC1-like population (Lin-CD56+CD94+CD16-CD127+) with impaired cytotoxicity in AML patients at diagnosis that is restored in patients who achieve remission [15]. A caveat to both of these studies is the sole use of surface markers to distinguish a conventional NK cell from an ILC1 or ILC1-like cell, and the phenotypic definition used in the latter study overlaps with CD56^bright^ NK cells found in circulation [72,73,74]. Traditionally, one of the fundamental differences between NK cells and ILC1s in both mice and humans is co-expression of EOMES along with T-BET among NK cells, whereas ILC1s only express T-BET. Greater clarity dissecting group 1 ILC heterogeneity in AML, and potentially other malignancies, could be achieved by combining surface immunophenotypes with the underlying transcription factor signatures of these populations. 

More recently, the inhibitory surface receptor, CD200R1, has been shown to be a selective surface marker for ILC1s in the murine liver and is notably absent on murine NK cells [71,72]. Interestingly, Coles et al. showed that expression of CD200 on human AML blasts is capable of inhibiting IFNγ secretion and reducing cytotoxicity via ligation of CD200R1 on human NK cells [26]. While the expression patterns of CD200R1 are still being studied in the context of human ILCs, it should be noted that this correlation may be the result of increased ILC1s in CD200^Hi^ AML, which would also corroborate the observation of reduced cytotoxicity which is commonly associated with an ILC1-like phenotype.

## 6. ILC2s: Potential Cancer Promoters?

ILC2s are typically associated with regulating allergic reactions and providing anti-helminthic immunity. Recent studies now show that ILC2s may also have pro-tumorigenic functions. One such study in AML revealed that mesenchymal stem cell-derived prostaglandin D2 (PGD2) is capable of activating ILC2s via the CRTH2 receptor to secrete IL-5, resulting in expansion of Treg populations and promoting HSPC proliferation [33]. This Treg expansion was associated with leukemia progression and worse survival in mouse models of AML. A similar observation has been made in acute promyelocytic leukemia (APL), a subset of AML in which tumor-derived PGD2 and NKp30-BH76 engagement activates ILC2s to secrete IL-13 that stimulates myeloid-derived suppressor cells (MDSC) [34]. Stimulation of MDSC’s has been widely shown to promote tumor growth [75]. Thus, therapeutic targeting of the PGD2-ILC2-Treg or -MDSC axis may serve as a viable treatment option for patients. In contrast, another study conducted by Trabanelli et al., showed that in the peripheral blood of untreated AML patients, there were no significant changes in ILC2 frequency, and they reported decreased levels of IL-5+IL-13 levels compared to healthy donors [32]. This discrepancy between the two studies in terms of observed ILC2 functions could be the result of tissue microenvironment differences, as the PGD2 producing mesenchymal cells were of bone marrow origin rather than peripheral blood. These seemingly contradictory observations underscore the importance of studying the relevant tissue microenvironments in a systemic disease like AML, as different tissues may have different biology. 

## 7. ILC3s: Guardians of the Gut?

To date there have been a limited number of studies investigating ILC3s in AML, although there is emerging evidence that ILC3s may have roles in determining prognosis post-chemotherapy and in combating GVHD pathology. 

One study reported a significant decrease in natural cytotoxicity receptor positive (NCR+) ILC3s but not NCR- ILC3s in the peripheral blood of treatment naïve AML patients [32]. This study defined ILC3s as Lin-CD127+CRTH2-CD117+NKp46+/− cells, which overlaps with immature NK cell and ILC precursor definitions found in circulation [13]. Interestingly, no detectable differences in IL-17A or IL-22, key ILC3-related cytokines, were observed between the circulating ILC compartment of healthy donors or AML patients in this study [32]. More recently, markers such as NKp44 have been shown to be more selective than CD117 in identifying ILC3 populations in humans [13], and healthy individuals do not normally contain detectable levels of NKp44+ cells in circulation [76]. This same study found that AML patients responding to standard chemotherapy had levels of Lin-CD127+CRTH2-CD117+NKp46+ cells comparable to normal donors while patients who failed to respond to therapy maintained reduced percentages, suggesting this population may have prognostic relevance in the setting of AML [32]. Separate studies have demonstrated that increased plasma IL-17 levels correlate with poor prognosis in AML [77,78], although the direct relationship between bona fide ILC3 and IL-17 in the setting of de novo AML is currently not known. Thus, more research is needed to fully elucidate the functional significance of ILC3 populations in AML not just in the periphery, but also in tissues.

In addition to their role in AML biology, ILC3s have also been studied in the reconstitution setting of post-induction chemotherapy regimens. Most patients with newly diagnosed AML undergo induction and consolidation chemotherapy, and for some, myeloablative conditioning followed by alloHSCT. A study conducted by Munneke et al. measured the reconstitution rates of different ILC subsets following induction chemotherapy and alloHSCT [35]. The results demonstrated that reconstitution of donor ILC1s (Lin-CD127+CRTH2-CD117-NKp44-), ILC2s (Lin-CD127+CRTH2+), and NKp44- ILC3s (Lin-CD127+CRTH2-CD117+NKp44-, discussed in preceding paragraph) was slow compared to the rate of monocyte and neutrophil recovery, while NKp44+ ILC3s (Lin-CD127+CRTH2-CD117+NKp44+) reconstituted more quickly relative to the other ILC subsets and at higher levels than found at steady-state. Although NKp44+ ILC3s are not normally found in the peripheral blood of healthy individuals, they were identified in the peripheral blood of AML patients receiving induction chemotherapy and patients who went on to receive alloHSCT. These ILC populations expressed activating markers such as CD69 and also homing receptors for the gut and skin, including α_4_β_7_ integrin, CCR6, CCR10, and CLA. The presence of these ILC populations following induction chemotherapy and/or alloHSCT were associated with a reduction in the incidence of GVHD. The authors speculated that these tissue-homing ILC populations protect against chemotherapy-induced epithelial damage in the gut, which likely contributes to the decreased incidence of GVHD. In mice, NCR+ ILC3s are known to participate in tissue repair in the gut and prevent bacterial translocation that may contribute to graft-versus-host disease (GVHD) pathology [79]. This was shown to be an IL-22 dependent mechanism. IL-22 has also been shown to protect against GVHD in an allogeneic HSCT mouse model [80]. Future studies will continue to dissect the role of these ILC populations in both AML biology and their predictive value in determining responses to therapy.

## 8. Mechanisms Leading to ILC Dysregulation in AML

**Figure 1:**
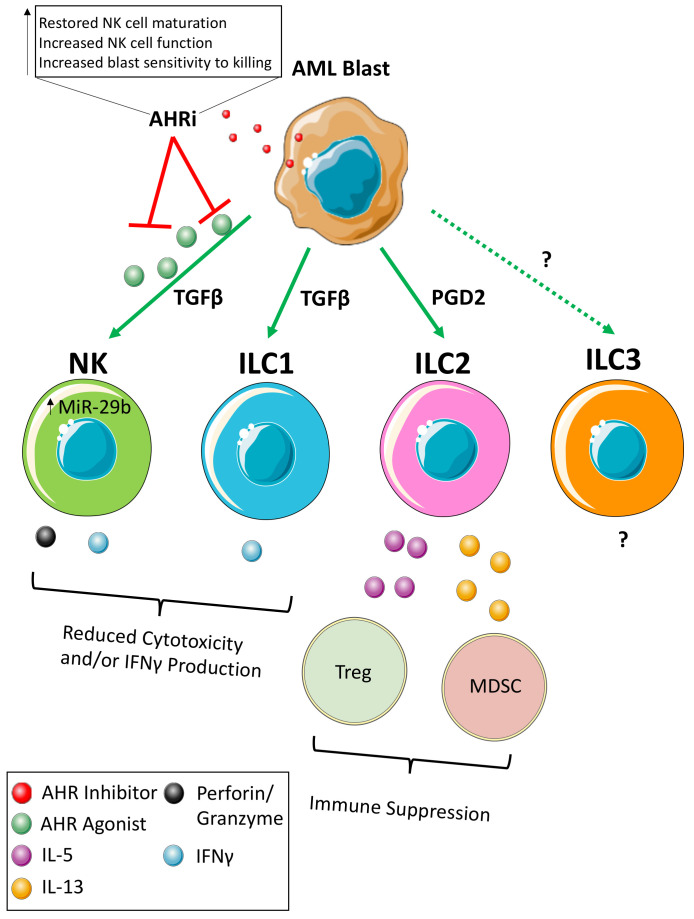
Schematic of ILC Dysregulation in AML. Working model of how AML blasts alter the function of innate lymphoid cell subsets.

The NK cells of AML patients and AML mouse models have been found to have a hypofunctional phenotype and appear less mature than age matched healthy donors. In mice, this is evidenced by decreased CD27+CD11b+NK cells [19] and in humans by a decrease in CD57+KIR+NK cells [21]. Part of the challenge has been to identify the exact mechanisms used by AML cells to alter NK/ILC development and function to allow for further targeted therapeutics (Figure 1). One such mechanism that has thus far been described involves the aryl hydrocarbon receptor (AHR), which is a ligand-activated transcription factor expressed both in NK cell developmental intermediates (NKDI) as well as AML blasts [20]. While the exact AHR ligand has yet to be identified, activation of AHR may yield an overall tumor-suppressive environment. For example, AHR agonists inhibit in vitro maturation of immature NK cells to cytotoxic effectors while AHR antagonists promote NK cell maturation [20,81]. Similarly, NKDIs co-cultured in the presence of AML blasts in a non-contact dependent mechanism produce NK cells with an immature and hypofunctional phenotype. At least part of the mechanism is thought to be due to AHR’s direct regulation of miR-29b, a known repressor of both T-BET and EOMES expression that are key lineage-defining transcription factors critical for the final maturation of NK cells [20,82,83]. Indeed, inhibition of miR-29b in NK cells in the setting of AML was capable of restoring their maturation [19]. While miR-29b negatively regulates NK cell development, it appears to possess tumor suppressive functions within AML blasts themselves [84,85], illustrating the cell-dependent role of miR-29b in AML. It was also discovered that antagonism of AHR with the small molecule inhibitor StemReginin 1 improves engraftment and expansion of CD34 cells into NK cells ex vivo, adding further rationale to study the therapeutic efficacy of AHR inhibition in AML therapeutics [86,87].

In addition to its role in NK cells, AHR is also important in regulating other ILC populations. Although AHR signaling has been shown to inhibit the development and normal function of NK cells, ILC3s are dependent on AHR for development, maintenance, and/or function [81,88]. Furthermore, evidence in mice suggests liver resident NK cells, which phenotypically overlap with ILC1s (DX5-CD49a+), rely on AHR signaling for maintenance [89]. ILC2 function has also been shown to be inhibited upon activation of the AHR pathway [90]. However, the consequences of these observation remain to be elucidated in AML in the context of AHR. It is possible that AML hijacks AHR signaling to inhibit NK cell development while promoting other ILC subsets that may be pro-tumorigenic. Further study is needed to determine the degree to which this occurs in AML. A final observation was also made that AML blasts pretreated with AHR antagonists are primed to NK cell-mediated killing, though the mechanism is still unknown [20]. Therefore, AHR is capable of both directly and indirectly regulating expression levels of genes important in the normal maturation of immune cells and has therapeutic potential.

While AHR activation reduces NK cell developmental potential in NKDIs [20], AHR activation in mature NK cells has the potential to increase their anticancer activity, highlighting the cell and stage-specific effects of this transcription factor [91,92]. Thus, the role of AHR in regulating ILC homeostasis is complex and dependent not only on the cell type being acted upon, but also on other microenvironment signals. Additionally, there is evidence of ligand-specific responses of AHR [93,94], underscoring the need to study how different AHR ligands affect downstream ILC responses at both steady-state and in the disease setting. One potentially relevant AHR agonist to study is the tryptophan degradation product kynurenine, which has been shown to play a role in the pathophysiology of glioblastoma [95]. Part of this catabolic pathway involves the enzyme IDO1, which is often hyperactive in solid tumors as well as in AML [96,97]. Identifying relevant AHR agonists in AML creates the possibility to both inhibit intracellular AHR signaling with small molecule inhibitors while also inhibiting production of the corresponding agonists. 

Additional NK cell development pathways have also been shown to be dysregulated in AML. Similar to AHR, NOTCH signaling is important to the development of ILC populations [98,99,100,101]. Not only is NOTCH signaling important for ILC development, activation of NOTCH has been shown to lead to AML blast apoptosis [102]. Therefore, inhibition of NOTCH signaling in AML blasts through expression of EGFL7 serves as a protective mechanism for the blasts to prevent their differentiation into a phenotypically mature cancer cell [103]. It remains unknown whether dysregulated NOTCH signaling in AML contributes to ILC dysregulation. Further investigation into how AML may dysregulate NOTCH signaling to skew ILC development may offer additional insight into how loss of mature NK cells occurs in AML patients. Future studies should also assess the clinical efficacy of EGFL7 as well as AHR inhibitors in AML.

In addition to direct inhibition of developmental pathways, AML may also affect the plasticity or conversion of seemingly mature ILC phenotypes with anticancer properties into pro-tumorigenic ILCs. Comprehensive reviews summarizing ILC plasticity have been previously published [104,105]. Notably, TGFβ-dependent conversion of mature NK cells with anti-cancer cell properties into non-cytotoxic ILC1s has been described in solid tumor mouse models [106]. Thus, the decrease in mature NK cells encountered in AML could be the result of not only skewed ILC development, but also conversion of NK cells into other non-cytolytic ILC subtypes. Additional investigation of the relative contributions of dysregulated ILC development and NK plasticity is needed, especially in hematologic malignancies like AML. 

While TGFβ has been extensively studied as a major immunomodulatory cytokine in the context of AML and other cancers, several other cytokines and signaling molecules could synergize with AHR to produce the immune phenotypes observed. For example, the pro-inflammatory molecule IL-1β has been heavily implicated in promoting ILC plasticity and conversion into other ILC subsets with altered function [104]. In the majority of cases, these altered functions promote cancer progression. Thus, further study is needed to fully elucidate the cytokine milieu in different cancers such as AML and the downstream impact this has on immune cell phenotypes and ultimately disease progression.

## 9. Conclusions

Overall, a common thread of immune dysregulation in AML involves loss of cytolytic effectors and gain of non-cytotoxic, immunomodulatory populations. As our mechanistic understanding of these phenotypic observations improve, our ability to target these pathways to restore the reservoir of cytolytic effectors will similarly advance. Moving forward, it will become important to study immune dysregulation in AML utilizing more sensitive omics-based approaches such as single cell sequencing of the ILC compartment. Because ILC biology is dependent on the presence or absence of surface markers, effector molecules, and transcription factors to define subsets, this -omics level approach is needed to define these cancer-associated ILC populations in a non-biased and concise manner. In addition, as our understanding of ILC plasticity continues to improve, future studies will need to identify the contribution of mature ILC conversion into other subtypes versus targeted developmental skewing of ILC precursors into specific ILC populations. By better understanding how these populations form and transform in AML, we will be better equipped to target these mechanisms pharmacologically to restore anti-cancer immunity and potentially improve clinical outcomes for AML patients. Future investigations should also draw parallels in NK/ILC dysfunction between different hematologic malignancies to look for commonalities and to determine whether these defects serve as central drivers promoting cancer immune evasion.

## Figures and Tables

**Table 1:** Summary of Innate Lymphoid Cell (ILC) Population Findings in Acute Myeloid Leukemia (AML).

Population	Species	Finding in AML	References
NK	Mouse	Developmental inhibition, loss of mature NK cells via AHR and miR-29b signaling	[19,20]
NK	Human	Inhibition of AHR sensitizes AML blasts to NK cell-mediated cytotoxicity and restores normal NK maturation	[20]
NK	Human	A hypomature peripheral NK phenotype (CD57-KIR-) correlates with worse overall survival in AML patients	[21]
NK	Human	A subset of AML patients has increased CD57+KIR+NK cells. Survival differences not assessed	[22]
NK	Human	CMV+ serostatus correlates with increased memory-like NK cell formation and longer periods of relapse-free survival but no change in overall survival	[23,24,25]
NK	Human	CD200^Hi^ AML patients have impaired NK cytotoxicity and lower IFNγ secretion	[26]
NK	Human	Increased expression of the inhibitory receptor NKG2A or decreased expression of activating receptors NKp30 and NKp46 show impaired NK cell function in AML patients which correlates with poor outcomes	[27,28,29,30,31]
ILC1	Human	Null definition ILC1s are enriched yet hypofunctional in AML patients	[32]
ILC1	Human	ILC1-like cells are hypofunctional in AML patients with cytotoxic function restored in AML patients who achieve remission	[15]
ILC2	Mouse/Human	Mesenchymal-derived PGD2 stimulates IL-5 secretion by ILC2s to promote Tregs, which in turn accelerate AML progression in mouse models	[33]
ILC2	Mouse/Human	APL-derived PGD2 stimulates IL-13 secretion by ILC2s which in turn support MDSC function	[34]
ILC3	Human	Decrease in NCR+ILC3 but not NCR-ILC3 in AML patient peripheral blood. No detectable differences in IL-17A or IL-22 levels. Treatment-responsive patients had restoration of NCR+ILC3s	[32]
ILC3	Human	Increased reconstitution rate of NKp44+ILC3 relative to other ILC populations. Higher expression of gut-homing receptors correlates with protection from GVHD	[35]

## References

[1] Dohner H., Weisdorf D.J., Bloomfield C.D. (2015). Acute Myeloid Leukemia. N. Engl. J. Med..

[2] Khwaja A., Bjorkholm M., Gale R.E., Levine R.L., Jordan C.T., Ehninger G., Bloomfield C.D., Estey E., Burnett A., Cornelissen J.J. (2016). Acute myeloid leukaemia. Nat. Rev. Dis Primers.

[3] DiNardo C.D., Pratz K., Pullarkat V., Jonas B.A., Arellano M., Becker P.S., Frankfurt O., Konopleva M., Wei A.H., Kantarjian H.M. (2019). Venetoclax combined with decitabine or azacitidine in treatment-naive, elderly patients with acute myeloid leukemia. Blood.

[4] Issa G.C., Kantarjian H.M., Xiao L., Ning J., Alvarado Y., Borthakur G., Daver N., DiNardo C.D., Jabbour E., Bose P. (2020). Phase II trial of CPX-351 in patients with acute myeloid leukemia at high risk for induction mortality. Leukemia.

[5] Döhner H., Estey E.H., Amadori S., Appelbaum F.R., Büchner T., Burnett A.K., Dombret H., Fenaux P., Grimwade D., Larson R.A. (2010). Diagnosis and management of acute myeloid leukemia in adults: Recommendations from an international expert panel, on behalf of the European LeukemiaNet. Blood.

[6] Lion E., Willemen Y., Berneman Z.N., Van Tendeloo V.F., Smits E.L. (2012). Natural killer cell immune escape in acute myeloid leukemia. Leukemia.

[7] Lambert J., Pautas C., Terré C., Raffoux E., Turlure P., Caillot D., Legrand O., Thomas X., Gardin C., Gogat-Marchant K. (2019). Gemtuzumab ozogamicin for de novo acute myeloid leukemia: Final efficacy and safety updates from the open-label, phase III ALFA-0701 trial. Haematologica.

[8] Ruggeri L., Capanni M., Urbani E., Perruccio K., Shlomchik W.D., Tosti A., Posati S., Rogaia D., Frassoni F., Aversa F. (2002). Effectiveness of donor natural killer cell alloreactivity in mismatched hematopoietic transplants. Science.

[9] Hsu K.C., Keever-Taylor C.A., Wilton A., Pinto C., Heller G., Arkun K., O’Reilly R.J., Horowitz M.M., Dupont B. (2005). Improved outcome in HLA-identical sibling hematopoietic stem-cell transplantation for acute myelogenous leukemia predicted by KIR and HLA genotypes. Blood.

[10] Ciurea S.O., Schafer J.R., Bassett R., Denman C.J., Cao K., Willis D., Rondon G., Chen J., Soebbing D., Kaur I. (2017). Phase 1 clinical trial using mbIL21 ex vivo-expanded donor-derived NK cells after haploidentical transplantation. Blood.

[11] Björklund A.T., Carlsten M., Sohlberg E., Liu L.L., Clancy T., Karimi M., Cooley S., Miller J.S., Klimkowska M., Schaffer M. (2018). Complete Remission with Reduction of High-Risk Clones following Haploidentical NK-Cell Therapy against MDS and AML. Clin. Cancer Res..

[12] Miller J.S., Soignier Y., Panoskaltsis-Mortari A., McNearney S.A., Yun G.H., Fautsch S.K., McKenna D., Le C., Defor T.E., Burns L.J. (2005). Successful adoptive transfer and in vivo expansion of human haploidentical NK cells in patients with cancer. Blood.

[13] Vivier E., Artis D., Colonna M., Diefenbach A., Di Santo J.P., Eberl G., Koyasu S., Locksley R.M., McKenzie A.N.J., Mebius R.E. (2018). Innate Lymphoid Cells: 10 Years On. Cell.

[14] Vivier E., Raulet D.H., Moretta A., Caligiuri M.A., Zitvogel L., Lanier L.L., Yokoyama W.M., Ugolini S. (2011). Innate or adaptive immunity? The example of natural killer cells. Science.

[15] Salome B., Gomez-Cadena A., Loyon R., Suffiotti M., Salvestrini V., Wyss T., Vanoni G., Ruan D.F., Rossi M., Tozzo A. (2019). CD56 as a marker of an ILC1-like population with NK cell properties that is functionally impaired in AML. Blood Adv..

[16] Cuff A.O., Sillito F., Dertschnig S., Hall A., Luong T.V., Chakraverty R., Male V. (2019). The Obese Liver Environment Mediates Conversion of NK Cells to a Less Cytotoxic ILC1-Like Phenotype. Front. Immunol..

[17] Artis D., Spits H. (2015). The biology of innate lymphoid cells. Nature.

[18] Chiossone L., Dumas P.Y., Vienne M., Vivier E. (2018). Natural killer cells and other innate lymphoid cells in cancer. Nat. Rev. Immunol..

[19] Morvan M.G., Lanier L.L. (2016). NK cells and cancer: You can teach innate cells new tricks. Nat. Rev. Cancer.

[20] Scoville S.D., Nalin A.P., Chen L., Chen L., Zhang M.H., McConnell K., Beceiro Casas S., Ernst G., Traboulsi A.A., Hashi N. (2018). Human AML activates the aryl hydrocarbon receptor pathway to impair NK cell development and function. Blood.

[21] Chretien A.S., Fauriat C., Orlanducci F., Galseran C., Rey J., Bouvier Borg G., Gautherot E., Granjeaud S., Hamel-Broza J.F., Demerle C. (2017). Natural Killer Defective Maturation Is Associated with Adverse Clinical Outcome in Patients with Acute Myeloid Leukemia. Front. Immunol..

[22] Chretien A.S., Granjeaud S., Gondois-Rey F., Harbi S., Orlanducci F., Blaise D., Vey N., Arnoulet C., Fauriat C., Olive D. (2015). Increased NK Cell Maturation in Patients with Acute Myeloid Leukemia. Front. Immunol..

[23] Elmaagacli A.H., Steckel N.K., Koldehoff M., Hegerfeldt Y., Trenschel R., Ditschkowski M., Christoph S., Gromke T., Kordelas L., Ottinger H.D. (2011). Early human cytomegalovirus replication after transplantation is associated with a decreased relapse risk: Evidence for a putative virus-versus-leukemia effect in acute myeloid leukemia patients. Blood.

[24] Foley B., Cooley S., Verneris M.R., Pitt M., Curtsinger J., Luo X., Lopez-Vergès S., Lanier L.L., Weisdorf D., Miller J.S. (2012). Cytomegalovirus reactivation after allogeneic transplantation promotes a lasting increase in educated NKG2C+ natural killer cells with potent function. Blood.

[25] Green M.L., Leisenring W.M., Xie H., Walter R.B., Mielcarek M., Sandmaier B.M., Riddell S.R., Boeckh M. (2013). CMV reactivation after allogeneic HCT and relapse risk: Evidence for early protection in acute myeloid leukemia. Blood.

[26] Coles S.J., Wang E.C., Man S., Hills R.K., Burnett A.K., Tonks A., Darley R.L. (2011). CD200 expression suppresses natural killer cell function and directly inhibits patient anti-tumor response in acute myeloid leukemia. Leukemia.

[27] Stringaris K., Sekine T., Khoder A., Alsuliman A., Razzaghi B., Sargeant R., Pavlu J., Brisley G., de Lavallade H., Sarvaria A. (2014). Leukemia-induced phenotypic and functional defects in natural killer cells predict failure to achieve remission in acute myeloid leukemia. Haematologica.

[28] Costello R.T., Sivori S., Marcenaro E., Lafage-Pochitaloff M., Mozziconacci M.J., Reviron D., Gastaut J.A., Pende D., Olive D., Moretta A. (2002). Defective expression and function of natural killer cell-triggering receptors in patients with acute myeloid leukemia. Blood.

[29] Fauriat C., Just-Landi S., Mallet F., Arnoulet C., Sainty D., Olive D., Costello R.T. (2007). Deficient expression of NCR in NK cells from acute myeloid leukemia: Evolution during leukemia treatment and impact of leukemia cells in NCRdull phenotype induction. Blood.

[30] Chretien A.S., Devillier R., Fauriat C., Orlanducci F., Harbi S., Le Roy A., Rey J., Bouvier Borg G., Gautherot E., Hamel J.F. (2017). NKp46 expression on NK cells as a prognostic and predictive biomarker for response to allo-SCT in patients with AML. Oncoimmunology.

[31] Chretien A.S., Fauriat C., Orlanducci F., Rey J., Borg G.B., Gautherot E., Granjeaud S., Demerle C., Hamel J.F., Cerwenka A. (2017). NKp30 expression is a prognostic immune biomarker for stratification of patients with intermediate-risk acute myeloid leukemia. Oncotarget.

[32] Trabanelli S., Curti A., Lecciso M., Salomé B., Riether C., Ochsenbein A., Romero P., Jandus C. (2015). CD127+ innate lymphoid cells are dysregulated in treatment naïve acute myeloid leukemia patients at diagnosis. Haematologica.

[33] Wu L., Lin Q., Ma Z., Chowdhury F.A., Mazumder M.H.H., Du W. (2020). Mesenchymal PGD(2) activates an ILC2-Treg axis to promote proliferation of normal and malignant HSPCs. Leukemia.

[34] Trabanelli S., Chevalier M.F., Martinez-Usatorre A., Gomez-Cadena A., Salomé B., Lecciso M., Salvestrini V., Verdeil G., Racle J., Papayannidis C. (2017). Tumour-derived PGD2 and NKp30-B7H6 engagement drives an immunosuppressive ILC2-MDSC axis. Nat. Commun..

[35] Munneke J.M., Björklund A.T., Mjösberg J.M., Garming-Legert K., Bernink J.H., Blom B., Huisman C., van Oers M.H., Spits H., Malmberg K.J. (2014). Activated innate lymphoid cells are associated with a reduced susceptibility to graft-versus-host disease. Blood.

[36] Ishikawa E., Tsuboi K., Saijo K., Harada H., Takano S., Nose T., Ohno T. (2004). Autologous natural killer cell therapy for human recurrent malignant glioma. Anticancer Res..

[37] Freud A.G., Becknell B., Roychowdhury S., Mao H.C., Ferketich A.K., Nuovo G.J., Hughes T.L., Marburger T.B., Sung J., Baiocchi R.A. (2005). A human CD34(+) subset resides in lymph nodes and differentiates into CD56bright natural killer cells. Immunity.

[38] Freud A.G., Yu J., Caligiuri M.A. (2014). Human natural killer cell development in secondary lymphoid tissues. Semin. Immunol..

[39] Yu J., Freud A.G., Caligiuri M.A. (2013). Location and cellular stages of natural killer cell development. Trends Immunol..

[40] Lim A.I., Li Y., Lopez-Lastra S., Stadhouders R., Paul F., Casrouge A., Serafini N., Puel A., Bustamante J., Surace L. (2017). Systemic Human ILC Precursors Provide a Substrate for Tissue ILC Differentiation. Cell.

[41] Boeck C.L., Amberger D.C., Doraneh-Gard F., Sutanto W., Guenther T., Schmohl J., Schuster F., Salih H., Babor F., Borkhardt A. (2017). Significance of Frequencies, Compositions, and/or Antileukemic Activity of (DC-stimulated) Invariant NKT, NK and CIK Cells on the Outcome of Patients With AML, ALL and CLL. J. Immunother..

[42] Epling-Burnette P.K., Bai F., Painter J.S., Rollison D.E., Salih H.R., Krusch M., Zou J., Ku E., Zhong B., Boulware D. (2007). Reduced natural killer (NK) function associated with high-risk myelodysplastic syndrome (MDS) and reduced expression of activating NK receptors. Blood.

[43] Zhang W., Xie X., Mi H., Sun J., Ding S., Li L., Liu H., Wang H., Fu R., Shao Z. (2018). Abnormal populations and functions of natural killer cells in patients with myelodysplastic syndromes. Oncol. Lett..

[44] Hahn C.N., Chong C.E., Carmichael C.L., Wilkins E.J., Brautigan P.J., Li X.C., Babic M., Lin M., Carmagnac A., Lee Y.K. (2011). Heritable GATA2 mutations associated with familial myelodysplastic syndrome and acute myeloid leukemia. Nat. Genet..

[45] Ostergaard P., Simpson M.A., Connell F.C., Steward C.G., Brice G., Woollard W.J., Dafou D., Kilo T., Smithson S., Lunt P. (2011). Mutations in GATA2 cause primary lymphedema associated with a predisposition to acute myeloid leukemia (Emberger syndrome). Nat. Genet..

[46] Mace E.M., Hsu A.P., Monaco-Shawver L., Makedonas G., Rosen J.B., Dropulic L., Cohen J.I., Frenkel E.P., Bagwell J.C., Sullivan J.L. (2013). Mutations in GATA2 cause human NK cell deficiency with specific loss of the CD56(bright) subset. Blood.

[47] Pizzolo G., Trentin L., Vinante F., Agostini C., Zambello R., Masciarelli M., Feruglio C., Dazzi F., Todeschini G., Chilosi M. (1988). Natural killer cell function and lymphoid subpopulations in acute non-lymphoblastic leukaemia in complete remission. Br. J. Cancer.

[48] Tratkiewicz J.A., Szer J. (1990). Loss of natural killer activity as an indicator of relapse in acute leukaemia. Clin. Exp. Immunol..

[49] Lowdell M.W., Craston R., Samuel D., Wood M.E., O’Neill E., Saha V., Prentice H.G. (2002). Evidence that continued remission in patients treated for acute leukaemia is dependent upon autologous natural killer cells. Br. J. Haematol..

[50] Dauguet N., Récher C., Demur C., Fournié J.J., Poupot M., Poupot R. (2011). Pre-eminence and persistence of immature natural killer cells in acute myeloid leukemia patients in first complete remission. Am. J. Hematol..

[51] Szczepanski M.J., Szajnik M., Welsh A., Foon K.A., Whiteside T.L., Boyiadzis M. (2010). Interleukin-15 enhances natural killer cell cytotoxicity in patients with acute myeloid leukemia by upregulating the activating NK cell receptors. Cancer Immunol. Immunother..

[52] Dolstra H., Roeven M.W.H., Spanholtz J., Hangalapura B.N., Tordoir M., Maas F., Leenders M., Bohme F., Kok N., Trilsbeek C. (2017). Successful Transfer of Umbilical Cord Blood CD34(+) Hematopoietic Stem and Progenitor-derived NK Cells in Older Acute Myeloid Leukemia Patients. Clin. Cancer Res..

[53] Tey S.K. (2014). Adoptive T-cell therapy: Adverse events and safety switches. Clin. Transl. Immunol..

[54] Zhao X.Y., Yu X.X., Xu Z.L., Cao X.H., Huo M.R., Zhao X.S., Chang Y.J., Wang Y., Zhang X.H., Xu L.P. (2019). Donor and host coexpressing KIR ligands promote NK education after allogeneic hematopoietic stem cell transplantation. Blood Adv..

[55] Boudreau J.E., Hsu K.C. (2018). Natural Killer Cell Education and the Response to Infection and Cancer Therapy: Stay Tuned. Trends Immunol..

[56] Verneris M.R., Miller J.S., Hsu K.C., Wang T., Sees J.A., Paczesny S., Rangarajan H., Lee D.A., Spellman S.R., Lee S.J. (2020). Investigation of donor KIR content and matching in children undergoing hematopoietic cell transplantation for acute leukemia. Blood Adv..

[57] Feins S., Kong W., Williams E.F., Milone M.C., Fraietta J.A. (2019). An introduction to chimeric antigen receptor (CAR) T-cell immunotherapy for human cancer. Am. J. Hematol..

[58] Halim L., Maher J. (2020). CAR T-cell immunotherapy of B-cell malignancy: The story so far. Ther. Adv. Vaccines Immunother..

[59] Cummins K.D., Gill S. (2019). Will CAR T cell therapy have a role in AML? Promises and pitfalls. Semin. Hematol..

[60] Tang X., Yang L., Li Z., Nalin A.P., Dai H., Xu T., Yin J., You F., Zhu M., Shen W. (2018). First-in-man clinical trial of CAR NK-92 cells: Safety test of CD33-CAR NK-92 cells in patients with relapsed and refractory acute myeloid leukemia. Am. J. Cancer Res..

[61] Denman C.J., Senyukov V.V., Somanchi S.S., Phatarpekar P.V., Kopp L.M., Johnson J.L., Singh H., Hurton L., Maiti S.N., Huls M.H. (2012). Membrane-bound IL-21 promotes sustained ex vivo proliferation of human natural killer cells. PLoS ONE.

[62] Vasu S., Bejanyan N., Devine S., Krakow E., Krakow E., Logan B., Luznik L., Ragon B.K., Barrett E., Shan J. (2019). BMT CTN 1803: Haploidentical Natural Killer Cells (CSTD002) to Prevent Post-Transplant Relapse in AML and MDS (NK-REALM). Blood.

[63] Liu E., Marin D., Banerjee P., Macapinlac H.A., Thompson P., Basar R., Nassif Kerbauy L., Overman B., Thall P., Kaplan M. (2020). Use of CAR-Transduced Natural Killer Cells in CD19-Positive Lymphoid Tumors. N. Engl. J. Med..

[64] Zhu H., Blum R.H., Bernareggi D., Ask E.H., Wu Z., Hoel H.J., Meng Z., Wu C., Guan K.L., Malmberg K.J. (2020). Metabolic Reprograming via Deletion of CISH in Human iPSC-Derived NK Cells Promotes In Vivo Persistence and Enhances Anti-tumor Activity. Cell Stem Cell.

[65] O’Sullivan T.E., Sun J.C., Lanier L.L. (2015). Natural Killer Cell Memory. Immunity.

[66] Romee R., Rosario M., Berrien-Elliott M.M., Wagner J.A., Jewell B.A., Schappe T., Leong J.W., Abdel-Latif S., Schneider S.E., Willey S. (2016). Cytokine-induced memory-like natural killer cells exhibit enhanced responses against myeloid leukemia. Sci. Transl. Med..

[67] Zhang H., Chua K.S., Guimond M., Kapoor V., Brown M.V., Fleisher T.A., Long L.M., Bernstein D., Hill B.J., Douek D.C. (2005). Lymphopenia and interleukin-2 therapy alter homeostasis of CD4+CD25+ regulatory T cells. Nat. Med..

[68] Leong J.W., Chase J.M., Romee R., Schneider S.E., Sullivan R.P., Cooper M.A., Fehniger T.A. (2014). Preactivation with IL-12, IL-15, and IL-18 induces CD25 and a functional high-affinity IL-2 receptor on human cytokine-induced memory-like natural killer cells. Biol. Blood Marrow Transplant..

[69] Cooley S., He F., Bachanova V., Vercellotti G.M., DeFor T.E., Curtsinger J.M., Robertson P., Grzywacz B., Conlon K.C., Waldmann T.A. (2019). First-in-human trial of rhIL-15 and haploidentical natural killer cell therapy for advanced acute myeloid leukemia. Blood Adv..

[70] Liu E., Tong Y., Dotti G., Shaim H., Savoldo B., Mukherjee M., Orange J., Wan X., Lu X., Reynolds A. (2018). Cord blood NK cells engineered to express IL-15 and a CD19-targeted CAR show long-term persistence and potent antitumor activity. Leukemia.

[71] Weizman O.E., Adams N.M., Schuster I.S., Krishna C., Pritykin Y., Lau C., Degli-Esposti M.A., Leslie C.S., Sun J.C., O’Sullivan T.E. (2017). ILC1 Confer Early Host Protection at Initial Sites of Viral Infection. Cell.

[72] Riggan L., Freud A.G., O’Sullivan T.E. (2019). True Detective: Unraveling Group 1 Innate Lymphocyte Heterogeneity. Trends Immunol..

[73] Romagnani C., Juelke K., Falco M., Morandi B., D’Agostino A., Costa R., Ratto G., Forte G., Carrega P., Lui G. (2007). CD56brightCD16- killer Ig-like receptor- NK cells display longer telomeres and acquire features of CD56dim NK cells upon activation. J. Immunol..

[74] Dulphy N., Haas P., Busson M., Belhadj S., Peffault de Latour R., Robin M., Carmagnat M., Loiseau P., Tamouza R., Scieux C. (2008). An unusual CD56(bright) CD16(low) NK cell subset dominates the early posttransplant period following HLA-matched hematopoietic stem cell transplantation. J. Immunol..

[75] Kumar V., Patel S., Tcyganov E., Gabrilovich D.I. (2016). The Nature of Myeloid-Derived Suppressor Cells in the Tumor Microenvironment. Trends Immunol..

[76] Hazenberg M.D., Spits H. (2014). Human innate lymphoid cells. Blood.

[77] Han Y., Ye A., Bi L., Wu J., Yu K., Zhang S. (2014). Th17 cells and interleukin-17 increase with poor prognosis in patients with acute myeloid leukemia. Cancer Sci..

[78] Kuen D.S., Kim B.S., Chung Y. (2020). IL-17-Producing Cells in Tumor Immunity: Friends or Foes?. Immune Netw..

[79] Sonnenberg G.F., Monticelli L.A., Alenghat T., Fung T.C., Hutnick N.A., Kunisawa J., Shibata N., Grunberg S., Sinha R., Zahm A.M. (2012). Innate lymphoid cells promote anatomical containment of lymphoid-resident commensal bacteria. Science.

[80] Hanash A.M., Dudakov J.A., Hua G., O’Connor M.H., Young L.F., Singer N.V., West M.L., Jenq R.R., Holland A.M., Kappel L.W. (2012). Interleukin-22 protects intestinal stem cells from immune-mediated tissue damage and regulates sensitivity to graft versus host disease. Immunity.

[81] Hughes T., Briercheck E.L., Freud A.G., Trotta R., McClory S., Scoville S.D., Keller K., Deng Y., Cole J., Harrison N. (2014). The transcription Factor AHR prevents the differentiation of a stage 3 innate lymphoid cell subset to natural killer cells. Cell Rep..

[82] Steiner D.F., Thomas M.F., Hu J.K., Yang Z., Babiarz J.E., Allen C.D., Matloubian M., Blelloch R., Ansel K.M. (2011). MicroRNA-29 regulates T-box transcription factors and interferon-γ production in helper T cells. Immunity.

[83] Smith K.M., Guerau-de-Arellano M., Costinean S., Williams J.L., Bottoni A., Mavrikis Cox G., Satoskar A.R., Croce C.M., Racke M.K., Lovett-Racke A.E. (2012). miR-29ab1 deficiency identifies a negative feedback loop controlling Th1 bias that is dysregulated in multiple sclerosis. J. Immunol..

[84] Garzon R., Heaphy C.E., Havelange V., Fabbri M., Volinia S., Tsao T., Zanesi N., Kornblau S.M., Marcucci G., Calin G.A. (2009). MicroRNA 29b functions in acute myeloid leukemia. Blood.

[85] Gong J.N., Yu J., Lin H.S., Zhang X.H., Yin X.L., Xiao Z., Wang F., Wang X.S., Su R., Shen C. (2014). The role, mechanism and potentially therapeutic application of microRNA-29 family in acute myeloid leukemia. Cell Death Differ..

[86] Boitano A.E., Wang J., Romeo R., Bouchez L.C., Parker A.E., Sutton S.E., Walker J.R., Flaveny C.A., Perdew G.H., Denison M.S. (2010). Aryl hydrocarbon receptor antagonists promote the expansion of human hematopoietic stem cells. Science.

[87] Roeven M.W., Thordardottir S., Kohela A., Maas F., Preijers F., Jansen J.H., Blijlevens N.M., Cany J., Schaap N., Dolstra H. (2015). The Aryl Hydrocarbon Receptor Antagonist StemRegenin1 Improves In Vitro Generation of Highly Functional Natural Killer Cells from CD34(+) Hematopoietic Stem and Progenitor Cells. Stem Cells Dev..

[88] Lee J.S., Cella M., McDonald K.G., Garlanda C., Kennedy G.D., Nukaya M., Mantovani A., Kopan R., Bradfield C.A., Newberry R.D. (2011). AHR drives the development of gut ILC22 cells and postnatal lymphoid tissues via pathways dependent on and independent of Notch. Nat. Immunol..

[89] Zhang L.H., Shin J.H., Haggadone M.D., Sunwoo J.B. (2016). The aryl hydrocarbon receptor is required for the maintenance of liver-resident natural killer cells. J. Exp. Med..

[90] Li S., Bostick J.W., Ye J., Qiu J., Zhang B., Urban J.F., Avram D., Zhou L. (2018). Aryl Hydrocarbon Receptor Signaling Cell Intrinsically Inhibits Intestinal Group 2 Innate Lymphoid Cell Function. Immunity.

[91] Shin J.H., Zhang L., Murillo-Sauca O., Kim J., Kohrt H.E., Bui J.D., Sunwoo J.B. (2013). Modulation of natural killer cell antitumor activity by the aryl hydrocarbon receptor. Proc. Natl. Acad. Sci. USA.

[92] Moreno-Nieves U.Y., Mundy D.C., Shin J.H., Tam K., Sunwoo J.B. (2018). The aryl hydrocarbon receptor modulates the function of human CD56(bright) NK cells. Eur. J. Immunol..

[93] Denison M.S., Nagy S.R. (2003). Activation of the aryl hydrocarbon receptor by structurally diverse exogenous and endogenous chemicals. Annu. Rev. Pharmacol. Toxicol..

[94] Murray I.A., Morales J.L., Flaveny C.A., Dinatale B.C., Chiaro C., Gowdahalli K., Amin S., Perdew G.H. (2010). Evidence for ligand-mediated selective modulation of aryl hydrocarbon receptor activity. Mol. Pharmacol..

[95] Adams S., Teo C., McDonald K.L., Zinger A., Bustamante S., Lim C.K., Sundaram G., Braidy N., Brew B.J., Guillemin G.J. (2014). Involvement of the kynurenine pathway in human glioma pathophysiology. PLoS ONE.

[96] Cheong J.E., Sun L. (2018). Targeting the IDO1/TDO2-KYN-AhR Pathway for Cancer Immunotherapy—Challenges and Opportunities. Trends Pharmacol. Sci..

[97] Fukuno K., Hara T., Tsurumi H., Shibata Y., Mabuchi R., Nakamura N., Kitagawa J., Shimizu M., Ito H., Saito K. (2015). Expression of indoleamine 2,3-dioxygenase in leukemic cells indicates an unfavorable prognosis in acute myeloid leukemia patients with intermediate-risk cytogenetics. Leuk Lymphoma.

[98] Kyoizumi S., Kubo Y., Kajimura J., Yoshida K., Hayashi T., Nakachi K., Moore M.A., van den Brink M.R.M., Kusunoki Y. (2017). Fate Decision Between Group 3 Innate Lymphoid and Conventional NK Cell Lineages by Notch Signaling in Human Circulating Hematopoietic Progenitors. J. Immunol..

[99] Mjösberg J., Bernink J., Peters C., Spits H. (2012). Transcriptional control of innate lymphoid cells. Eur. J. Immunol..

[100] Perchet T., Petit M., Banchi E.G., Meunier S., Cumano A., Golub R. (2018). The Notch Signaling Pathway Is Balancing Type 1 Innate Lymphoid Cell Immune Functions. Front. Immunol..

[101] Nalin A.P., Kowalski J.J., Sprague A.C., Schumacher B.K., Gerhardt A.G., Youssef Y., Vedantam K.V., Zhang X., Siebel C.W., Mace E.M. (2020). Notch Regulates Innate Lymphoid Cell Plasticity during Human NK Cell Development. J. Immunol..

[102] Kannan S., Sutphin R.M., Hall M.G., Golfman L.S., Fang W., Nolo R.M., Akers L.J., Hammitt R.A., McMurray J.S., Kornblau S.M. (2013). Notch activation inhibits AML growth and survival: A potential therapeutic approach. J. Exp. Med..

[103] Bill M., Pathmanathan A., Karunasiri M., Shen C., Burke M.H., Ranganathan P., Papaioannou D., Zitzer N.C., Snyder K., LaRocco A. (2020). EGFL7 Antagonizes NOTCH Signaling and Represents a Novel Therapeutic Target in Acute Myeloid Leukemia. Clin. Cancer Res..

[104] Bal S.M., Golebski K., Spits H. (2020). Plasticity of innate lymphoid cell subsets. Nat. Rev. Immunol..

[105] Bald T., Wagner M., Gao Y., Koyasu S., Smyth M.J. (2019). Hide and seek: Plasticity of innate lymphoid cells in cancer. Semin. Immunol..

[106] Gao Y., Souza-Fonseca-Guimaraes F., Bald T., Ng S.S., Young A., Ngiow S.F., Rautela J., Straube J., Waddell N., Blake S.J. (2017). Tumor immunoevasion by the conversion of effector NK cells into type 1 innate lymphoid cells. Nat. Immunol..

